# Optimizing the Extraction of the Polyphenolic Fraction from Defatted Strawberry Seeds for Tiliroside Isolation Using Accelerated Solvent Extraction Combined with a Box–Behnken Design

**DOI:** 10.3390/molecules29133051

**Published:** 2024-06-27

**Authors:** Magdalena Wójciak, Barbara Mazurek, Weronika Wójciak, Dorota Kostrzewa, Magdalena Żuk, Mariusz Chmiel, Tomasz Kubrak, Ireneusz Sowa

**Affiliations:** 1 Department of Analytical Chemistry, Medical University of Lublin, Chodźki 4a, 20-093 Lublin, Poland; weronikawojciak01@gmail.com (W.W.); magdalena.zu25@gmail.com (M.Ż.); ireneusz.sowa@umlub.pl (I.S.); 2 Analytical Department, Łukasiewicz Research Network—New Chemical Syntheses Institute, Aleja Tysiąclecia Państwa Polskiego 13a, 24-110 Puławy, Poland; barbara.mazurek@ins.lukasiewicz.gov.pl (B.M.); dorota.kostrzewa@ins.lukasiewicz.gov.pl (D.K.); mariusz.chmiel@ins.lukasiewicz.gov.pl (M.C.); 3 Department of Biochemistry and General Chemistry, Medical College, University of Rzeszów, 2A Kopisto St., 35-959 Rzeszów, Poland; tkubrak@ur.edu.pl

**Keywords:** *Fragaria ananassa*, polyphenols, tiliroside, ASE, defatted seeds

## Abstract

Tiliroside is a natural polyphenolic compound with a wide range of biological activity, and defatted strawberry seeds are its rich source. The goal of this study was to optimize accelerated solvent extraction (ASE) conditions, including temperature, solvent composition, and the number of extraction cycles, using Box–Behnken design to maximize the yield of tiliroside. UPLC-DAD-MS was applied to investigate the polyphenolic composition of the extracts, and preparative liquid chromatography (pLC) was used for isolation. All obtained mathematical models generally showed an increase in the efficiency of isolating polyphenolic compounds with an increase in temperature, ethanol content, and the number of extraction cycles. The optimal established ASE conditions for tiliroside were as follows: a temperature of 65 °C, 63% ethanol in water, and four extraction cycles. This allowed for the obtainment of a tiliroside-rich fraction, and the recovery of isolated tiliroside from plant material reached 243.2 mg from 100 g. Our study showed that ASE ensures the isolation of a tiliroside-rich fraction with high effectiveness. Furthermore, defatted strawberry seeds proved to be a convenient source of tiliroside because the matrix of accompanying components is relatively poor, which facilitates separation.

## 1. Introduction

**Figure 1:**
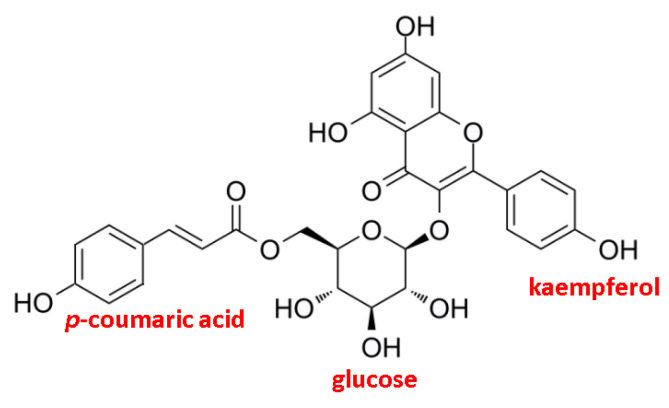
Chemical structure of tiliroside—kaempferol-3-O-β-D-(6″-E-p-coumaroyl)-glucopyranoside.

Tiliroside is a natural polyphenolic compound belonging to the derivatives of kaempferol, which contains glucose and a coumaroyl substituent attached to the aglycone ring (Figure 1).

Numerous scientific studies indicate that tiliroside possesses a wide range of biological activities, including a positive impact on the cardiovascular and nervous systems [1,2], antiobesity and antidiabetic properties [3,4,5], as well as anti-inflammatory action [6,7]. It also inhibits the proliferation of different types of cancer cells [8,9] and shows significant antioxidant effects observed in both chemical tests and investigations using biological systems [7,9]. Furthermore, it acts beneficially on skin cells by protecting them from oxidative stress and inflammation, decreasing tyrosinase activity, enhancing the ceramide layer, and thereby preventing moisture loss [7,10,11,12]. Taking into account the aforementioned properties, it can be considered a valuable dietary supplement and cosmetic additive.

Tiliroside was first isolated from *Rosa canina*, and to date, it has been found in 172 species, mostly from the Rosaceae and Malvaceae families. It is most abundant in flowers, followed by leaves, with the lowest concentration in roots and fruits [13]. For example, a significant amount of tiliroside was detected in the aerial parts of some *Potentilla* spp. [14] and in the flowers of *Gossypium hirsutum* [15], *Rosa rugose*, *Althaea rosea* [16], *Tilia cordata,* and *T. platyphyllos* [16,17,18].

The initial step in isolating tiliroside from plant material is solid–liquid extraction (SLE) using organic solvents, mostly ethanol or methanol with varying water content, followed by a partition through liquid–liquid extraction and preparative chromatography [15,19,20,21]. The extraction process can be assisted by ultrasound or microwave to enhance extraction efficiency and decrease processing time [21,22,23]. In our study, accelerated solvent extraction (ASE) was used to facilitate the extraction of the polyphenolic fraction, which was then applied to isolate tiliroside from the plant matrix. This technique offers several advantages, such as high effectiveness, precise control over extraction parameters, and automatization, resulting in improved reproducibility [24,25,26]. Moreover, samples of plant material placed in reaction cells do not have access to light and oxygen during the extractions, which limits the degradation processes. Increased temperature and/or pressure reduce the viscosity of the solvent, facilitating its penetration between the matrix particles and increasing its capacity to dissolve polyphenols [27].

The goal of this study was to optimize ASE conditions, including temperature, solvent composition, and the number of extraction cycles using Box–Behnken design to maximize the yield of tiliroside in a total phenolic fraction, which was further isolated using preparative liquid chromatography (pLC). BBD is suitable for examining the influence of any number of selected parameters and for process optimization [28,29]. Moreover, it facilitates the acquisition of information on possible interactions between the evaluated parameters. It enables the creation of a mathematical model based on data obtained from several experiments without the need to perform dozens of them.

Defatted strawberry seeds were used as research material because the reports showed that they are a quite rich reservoir of tiliroside with a content in the range of 130 to 184.0 mg/100 g [22,30]. Additionally, this is a convenient source for obtaining tiliroside, as the amount of fatty acids and other lipid components in the matrix is highly reduced, and the polyphenolic profile is modest, facilitating targeted isolation. Furthermore, the utilization of fruit and vegetable byproducts generated during the processing for the food industry is an important trend from the perspective of a “zero waste” strategy.

## 2. Results and Discussion

### 2.1. Optimization of Conditions for Accelerated Solvent Extraction (ASE)

Accelerated solvent extraction (ASE) was used to isolate polyphenolic fractions from plant material. In this technique, it is possible to use a wide range of solvents. However, taking into account current trends in environmental protection and with a view to possible future applications in cosmetics or pharmaceutical products, two solvents considered neutral, namely water and ethanol, were used in our work.

The temperature, solvent ratio, and the number of extraction cycles were investigated as the main factors influencing the composition of the extract and the efficiency of the process [31,32,33,34,35]. The ranges of parameters were selected based on a literature review and previous preliminary research. The indicated independent variables are factors that can be modified in the ASE system. In turn, the effect of pressure is to maintain the solvents as liquids even above their atmospheric boiling points [28]. The pressures used in ASE are well above the thresholds required to maintain the solvents in their liquid states, so pressure adjustments for changing solvents are not required. Changing the pressure has very little impact on analyte recovery and is not considered a critical experimental parameter [35]. Therefore, ASE extractions were performed at a constant pressure of 1500 psi. The situation is different in the case of temperature. Operation at high temperatures may worsen extract quality if the extraction is too long [36]. Due to the possibility of the thermal degradation of phenolic compounds, a temperature of 60–70 °C is usually not exceeded, as suggested by Rajha et al. [37]. Some authors, such as Volf et al. [38], suggest a lower temperature range of 40–50 °C, while others recommend much higher temperatures [39,40,41,42]. In this study, the range of variables was carefully chosen to include all parameter spaces and to obtain well-fitting models.

The complete matrix of the experimental plan and the results of quantitative analysis of the main metabolites using the UPLC-DAD method in ASE extract obtained during the implementation of the Box–Behnken design is shown in Table 1. In addition to the target metabolite (tiliroside), other phenolic components of defatted strawberry seeds were also monitored to assess the overall effectiveness of the polyphenol extraction process.

#### 2.1.1. Extraction Yield

The yield value of performed ASE extraction processes of defatted strawberry seeds ranged from 4.67% to 6.22%. Statistical analysis shows that the extraction yield depends on temperature (X_1_), solvent ratio (X_2_), and extraction cycles (X_3_). In the first modeling approach, a quadratic equation was selected (yield = 5.4733 + 0.1813X_1_ + 0.4888X_2_ + 0.1138X_3_ − 0.0562X_1_X_2_ − 0.0037X_1_X_3_ + 0.0637X_2_X_3_ − 0.0110X_1_2 − 0.0235X_2_^2^ + 0.0190X_3_^2^), for which, however, the quadratic terms and interactions between the parameters turned out to be insignificant (*p* > 0.10). Moreover, the predicted R^2^ was only 0.5420. It was found that quadratic relations and interactions between parameters are not significant. Therefore, the linear model for extraction yield was used. This model is significant (*p* < 0.0001), and the lack of fit of the model is insignificant (*p* = 0.2700). Values of the coefficient of determination R^2^, adjusted coefficient of determination R^2^, and predicted coefficient of determination R^2^ are high and are 0.9549, 0.9426, and 0.9065, respectively.

**Figure 2:**
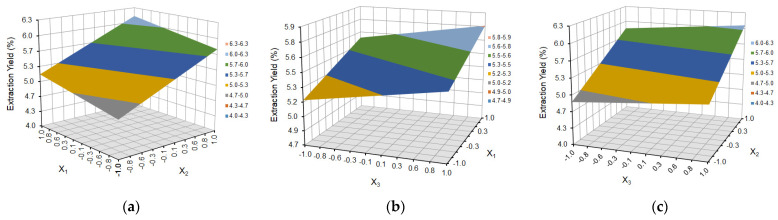
Response surface plot for the extraction yield as a function of (**a**) temperature (X_1_) and solvent ratio (X_2_), where X_3_ = 0; (**b**) temperature (X_1_) and extraction cycles (X_3_), where X_2_ = 0; and (**c**) solvent ratio (X_2_) and extraction cycles (X_3_), where X_1_ = 0.

Figure 2 shows the surface plot for extraction yield as a function of variables X_1_ (temperature), X_2_ (solvent ratio), and X_3_ (extraction cycles).

Analysis of the response surface graphs (Figure 2) and the fitted model equation indicate that the higher the extraction temperature and the higher the number of extraction cycles, the more the extraction yield increases. As the temperature increases, the viscosity of the solvent is reduced, thereby increasing its ability to wet the matrix and solubilize the target analytes. The added thermal energy also assists in breaking analyte–matrix bonds and encourages analyte diffusion from the matrix surface. The choice of temperature may also affect selectivity [28,35,43]. At higher temperatures, the extraction is less selective. Lowering the temperature will make ASE more selective, but the recovery of analytes can decrease unless the extraction time is increased. Increasing extraction cycles allows for the introduction of fresh solvents during the ASE extraction process, which helps maintain a favorable extraction balance. In addition, static cycles are useful for samples with varying concentrations of analytes, allowing for their more complete extraction, as well as for samples with difficult-to-penetrate matrices (e.g., plant seeds). If extraction at a lower temperature (<75 °C) is desired, as is the case with the extraction of compounds with low thermal stability, such as polyphenolic compounds, several static cycles should be used instead of one to increase the solubility of the analytes. This helps compensate for the lower amount of solvent introduced during the heating stage and maintains the specified pressure level [28]. The yield values are also higher both at a higher process temperature and at a higher water content in the water/ethanol ratio in the selected solvent system. This is because the ASE selectivity is decreased when using more polar solvents and more components are extracted (not necessarily those determined in the study), which collectively affects the efficiency of the extraction process.

**Figure 3:**
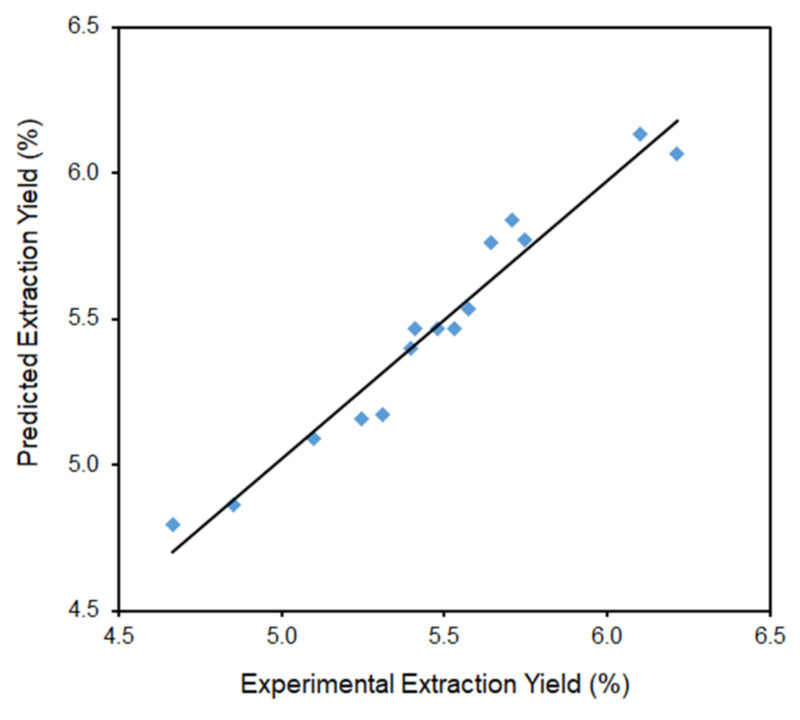
Comparison of the experimental results with the results predicted according to the developed mathematical models for extraction yield (y = 0.9549x + 0.2465).

Figure 3 shows the relationship between the predicted extraction yield of defatted strawberry seeds and the experimental data.

The final form/equation of the mathematical model for the extraction yield as a function of coded variables is

yield = 5.47 + 0.18X_1_ + 0.49X_2_ + 0.11X_3_ (1)

#### 2.1.2. Individual Polyphenol Yield of Defatted Strawberry Seeds

Based on the experimental data presented in Table 2, the statistical analyses of the second-order polynomial equation for the yield of ferulic acid derivative (Fad), *p*-coumaric acid (*p*-CA), 3-glucoside kaempferol (3gluK), ellagic acid (EA), and tiliroside were carried out. The results of statistical analysis for full quadratic models are presented in Table 2.

The significance of the equation parameters for the response variable was assessed by the probability (p) of 0.1. The statistically found non-significant terms (*p* > 0.1) were dropped from the initial models (not counting those required to support hierarchy). The adequacy and predictability of the mathematical models were considered according to the coefficient of determination R^2^, adjusted and predicted coefficient of determination R^2^, and the lack of fit. Adjusted R^2^, a modified version of R^2^, increases precision and reliability by taking into account the effects of additional independent variables that tend to distort the results of R^2^ measurements. The predicted R^2^, as opposed to the adjusted R-squared, is used to indicate how well the regression model predicts responses to new observations.

The statistical analysis indicated that the proposed quadratic models for ferulic acid derivative, *p*-coumaric acid, kaempferol 3-glucoside, ellagic acid, and tiliroside were statistically significant with good determination coefficient R^2^ (0.9704, 0.9598, 0.9747, 0.9963, and 0.9910). At the same time, it was observed that the models contained some terms that were statistically not significant. Moreover, some predicted R^2^ values were low (0.6131, 0.5167, 0.6450), and the lack of fit for tiliroside was significant (*p* = 0.0275). Therefore, the proposed models were reduced by removing insignificant coefficients (*p* > 0.10) using the step-by-step method (not counting those required to support hierarchy). The results of the statistical analysis for the reduced mathematical models are presented in Table 3.

The presented procedure has been used in the literature for model extraction processes [29,34,40]. The step-by-step reduction of statistically insignificant factors resulted in an increase in the predicted R^2^. The adjusted R^2^ and predicted R^2^ should be within 20% to maintain good agreement, as suggested by Owolabi et al. [44].

In the case of reduced models for ferulic acid derivative, *p*-coumaric acid, kaempferol 3-glucoside, and ellagic acid, a very low value of the *p* parameter was obtained (*p* ≤ 0.0002), confirming that the developed models are very highly statistically significant. Furthermore, the results showed that the lack of fit was statistically not significant (*p* > 0.05). The non-significant lack of fit for models suggests the adequacy of models to explain data in the region of experimentation. Also, the reduction of statistically insignificant components resulted in an improvement of the R^2^ predicted coefficient for ferulic acid derivative, *p*-coumaric acid, kaempferol 3-glucoside, and ellagic acid (0.8479, 0.8451, 0.8044, 0.9305, 0.9611). Therefore, the obtained values indicate that the reduced models can be used to predict the yield of the selected polyphenolic compounds in the given range of the independent variables.

In the case of tiliroside yield, despite the reduction of non-significant coefficients, the lack of fit was still statistically significant (*p* = 0.045 < 0.05). To obtain a better model, transformations of the results were used. As a result of the applied transformation (ln (Y)), a highly statistically significant model was obtained with very high coefficients of determination R^2^, adjusted R^2^, and predicted R^2^ and an insignificant lack of fit (*p* = 0.0842) (Table 3). Statistical analysis of regression coefficients indicates significant model terms for the yield of individual polyphenols.

For ferulic acid derivative, *p*-coumaric acid, kaempferol 3-glucoside, and ellagic acid, the significant model terms are the linear terms of temperature (X_1_) and the solvent ratio (X_2_) and the quadratic term of the solvent ratio (X_2_^2^). The linear expression of the number of static cycles (X_3_) is only relevant for *p*-coumaric and ellagic acids. However, for the ferulic acid derivative, the main effect of the number of static cycles (X3) was added to support the hierarchy. For ferulic acid derivative and *p*-coumaric acid expressions defining interaction with temperature are important (X_1_X_2_ and X_1_X_3_, respectively), and for ellagic acid, the interaction of the solvent ratio and the number of extraction cycles (X_2_X_3_) is important. The linear term of the extraction cycles (X_3_), all interactions of the studied independent variables, quadratic term of temperature (X_1_^2^), and quadratic term of extraction cycles (X_3_^2^) were regarded as statistically insignificant for the yield of kaempferol 3-glucoside. But, for the ferulic acid derivative, the quadratic term of temperature (X_1_^2^) and quadratic term of extraction cycles (X_3_^2^) are statistically significant. In the case of tiliroside, the significant model terms are the linear terms of temperature (X_1_), the solvent ratio (X_2_), and the number of static cycles (X_3_). Also, the significant terms of the model are the interaction of the solvent ratio and the number of extraction cycles (X_2_X_3_), the quadratic term of temperature (X_1_^2^), and the quadratic term of the solvent ratio (X_2_^2^).

**Figure 4:**
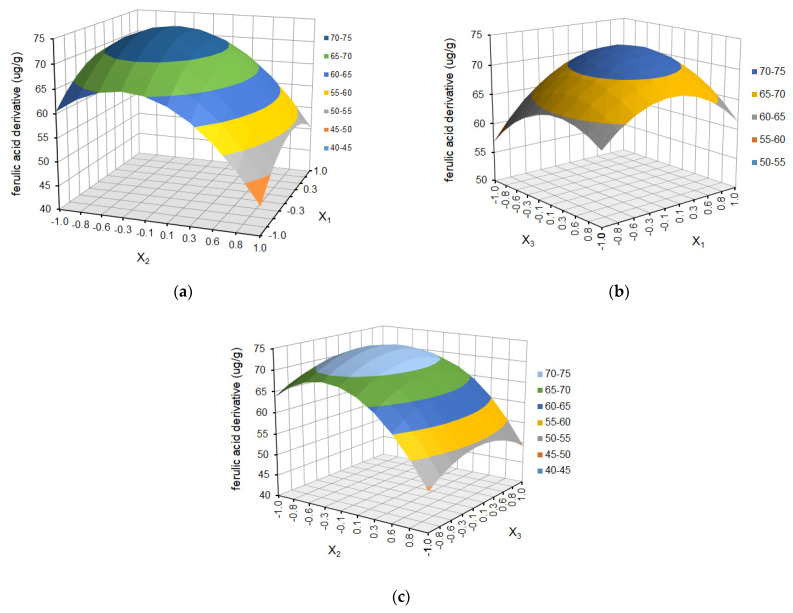
Response surface plot for ferulic acid derivative as a function of (**a**) temperature (X_1_) and solvent ratio (X_2_), where X_3_ = 0; (**b**) temperature (X_1_) and extraction cycles (X_3_), where X_2_ = 0; and (**c**) solvent ratio (X_2_) and extraction cycles (X_3_), where X_1_ = 0.

**Figure 5:**
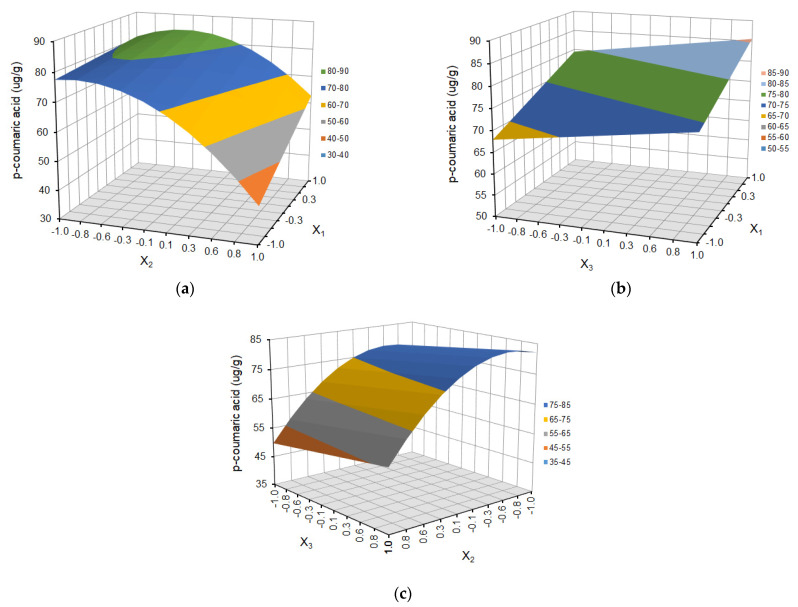
Response surface plot for *p*-coumaric acid as a function of (**a**) temperature (X_1_) and solvent ratio (X_2_), where X_3_ = 0; (**b**) temperature (X_1_) and extraction cycles (X_3_), where X_2_ = 0; and (**c**) solvent ratio (X_2_) and extraction cycles (X_3_), where X_1_ = 0.

**Figure 6:**
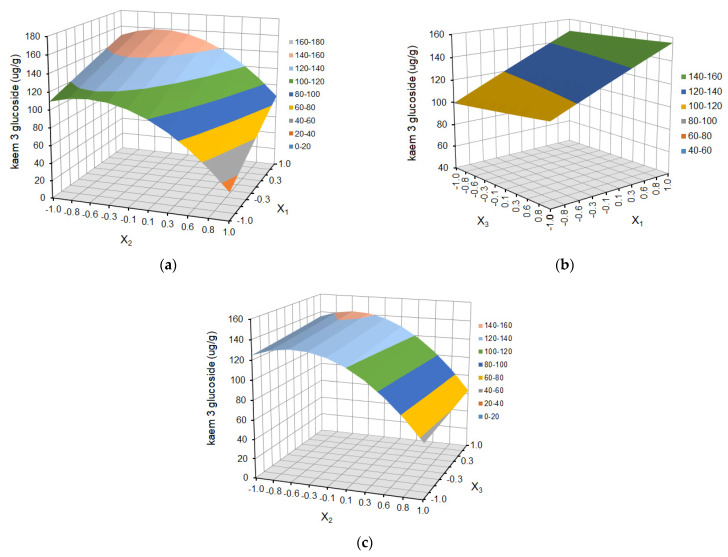
Response surface plot for kaempferol 3-glucoside as a function of (**a**) temperature (X_1_) and solvent ratio (X_2_), where X_3_ = 0; (**b**) temperature (X_1_) and extraction cycles (X_3_), where X_2_ = 0; and (**c**) solvent ratio (X_2_) and extraction cycles (X_3_), where X_1_ = 0.

**Figure 7:**
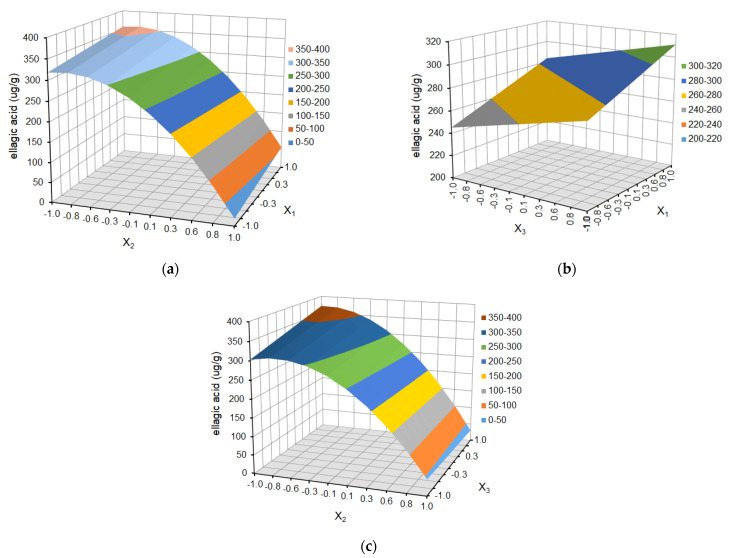
Response surface plot for ellagic acid as a function of (**a**) temperature (X_1_) and solvent ratio (X_2_), where X_3_ = 0; (**b**) temperature (X_1_) and extraction cycles (X_3_), where X_2_ = 0; and (**c**) solvent ratio (X_2_) and extraction cycles (X_3_), where X_1_ = 0.

**Figure 8:**
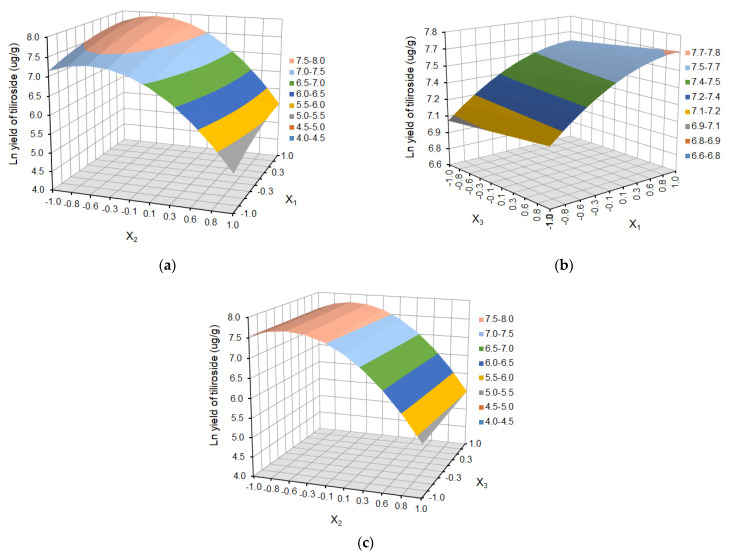
Response surface plot after (ln (Y)) transformation for tiliroside as a function of (**a**) temperature (X_1_) and solvent ratio (X_2_), where X_3_ = 0; (**b**) temperature (X_1_) and extraction cycles (X_3_), where X_2_ = 0; and (**c**) solvent ratio (X_2_) and extraction cycles (X_3_), where X_1_ = 0.

The effect of temperature (X_1_), solvent ratio (X_2_), and extraction cycles (X_3_) on the dependent variables (the yield of ferulic acid derivative, *p*-coumaric acid, kaempferol 3-glucoside, ellagic acid, and tiliroside) is shown as response surface plots in Figure 4, Figure 5, Figure 6, Figure 7 and Figure 8, respectively.

The choice of the appropriate temperature is an important factor in the optimization of the extraction process since it has a significant impact on the effectiveness of the process and the overall quality of the extract [36,38]. As is shown in Figure 4a, Figure 5a, Figure 6a, Figure 7a and Figure 8a and Figure 4b, Figure 5b, Figure 6b, Figure 7b and Figure 8b, the yield of selected compounds increases with the temperature increase from 45 to 65 °C. When the temperature increased, the viscosity of the solvent decreased, and the molecular movement speed in the matrix increased gradually. The solubility and the extraction rate of polyphenols increased [34]. When the temperature increased, the viscosity of the solvent decreased, and the molecular movement speed in the matrix increased gradually. The solubility and the extraction rate of polyphenols increased. For ferulic acid derivative as the only compound, the graph takes the shape of a dome, and the maximum values are obtained in the temperature area of 53–60 °C. When the temperature was more than 60 °C, the yield of ferulic acid derivative began to reduce. For the remaining compounds, the graphs are a slightly inclined plane.

In the case of solvent ratio (Figure 4a, Figure 5a, Figure 6a, Figure 7a and Figure 8a and Figure 4c, Figure 5c, Figure 6c, Figure 7c and Figure 8c), as the ethanol content in the water/ethanol configuration increases, the polyphenolic yield increases, in general. The ethanol concentration determines the polarity of the extraction solvent, which determines the hydrophobic force and the hydrogen bond bonding strength of the target components in the solvent, which affects the solubility and extraction rate of the target components. Following the theory of similarity and inter-miscibility, the more similar the polarity of solvent and solute, the faster the dissolution of solute from plant cells.

However, in several of the compounds discussed (ferulic acid derivative, *p*-coumaric acid, kaempferol 3-glucoside, and tiliroside), the yield began to decrease when the ethanol content exceeded approximately 60% for the ferulic acid derivative and *p*-coumaric acid and 65% for the kaempferol 3-glucoside and tiliroside, respectively.

According to the literature review, this may be due to the fact that water and ethanol in low concentrations can easily penetrate cell membranes, while ethanol in high concentrations can cause protein denaturation, preventing the dissolution of, among others, polyphenols and influencing the extraction rate [45,46]. Moreover, according to Hu et al. (2016), the high water content causes much matrix swelling, which facilitates the penetration of the solvent into the material. However, an increased concentration of ethanol and a greater water content in the solvent leads to less swelling of the material, which may have a negative impact on extraction efficiency [33,34].

Examples of other studies also showed that increasing the share of ethanol in the extraction mixture only to a certain level increased the content of polyphenols in the obtained product. Cacace and Mazza studied the impact of ethanol concentration on the optimal extraction of polyphenols from blackcurrants. They found that total phenolics increased with ethanol concentration up to a maximum of about 60% and then decreased with a further increase in solvent concentration irrespective of the solvent-to-solid ratio [47].

The number of extraction cycles is also important because multiple extraction cycles may result in the complete extraction of targeted compounds. In the case of dependence and the yield of polyphenols regarding the temperature (X_1_) and the number of cycles (X_3_) (Figure 4b, Figure 5b, Figure 6b, Figure 7b, Figure 8b + Figure 4c, Figure 5c, Figure 6c, Figure 7c, Figure 8c), with an increase in the number of cycles (X_3_), there was an increase in the yield of the defined compounds at a constant temperature value (X_1_). This is related to the extraction time and, therefore, to the contact time of the solvent with the extracted material. In the present study, the number of extraction cycles significantly influences the yield of *p*-coumaric and ellagic acid, whereas, in the case of ferulic acid derivative, the interaction effects between temperature and number of cycles have a significant effect on yield (Table 3). When the temperature was more than 60 °C, and the number of extraction cycles was more than 3, the yield of ferulic acid derivative began to reduce.

**Figure 9:**
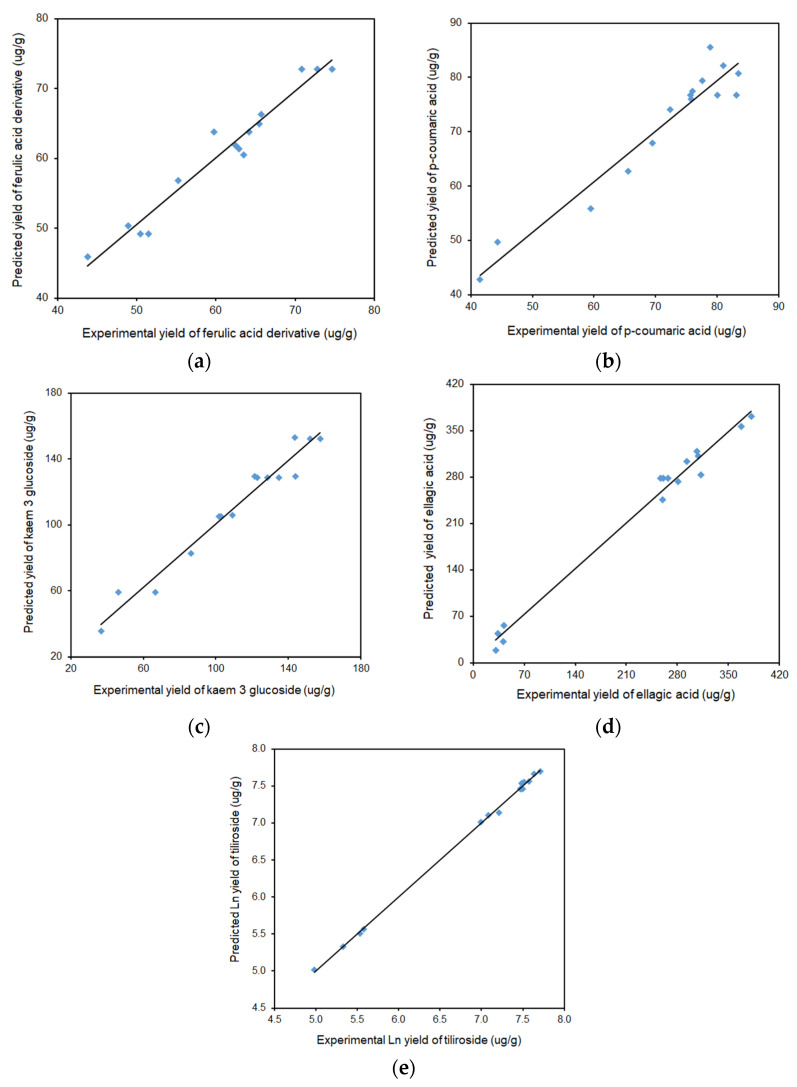
Comparison of the experimental results with the results predicted according to the developed mathematical models for (**a**) ferulic acid derivative (y = 0.9617x + 2.3313), (**b**) *p*-coumaric acid (y = 0.9294x + 5.0086), (**c**) kaempferol 3 glucoside (y = 0.9629x + 4.0935), (**d**) ellagic acid (y = 0.9864x + 3.1241), and (**e**) tiliroside (y = 0.9989x + 0.0073).

Figure 9a–e shows the relationship between the predicted and experimental data for yield of ferulic acid derivative, *p*-coumaric acid, kaempferol 3-glucoside, ellagic acid, and tiliroside, respectively.

As can be seen in Figure 9, the predicted values are well correlated with the experimental values; hence, all data points cluster near the determined curves. No significantly scattered points are observed, and the graph clearly shows that the obtained experimental values closely coincide with those developed by the model. The presented regression curves pass through many evenly distributed test points. Probably, the range of adopted research parameters is slightly better selected in relation to the other presented compounds rather than for ellagic acid and tiliroside, hence the concentration of points in the presented graphs at the beginning and end of the curve (without their distribution in the middle part—Figure 9d,e).

The final reduced mathematical models for response variables (yield of defined polyphenols) in the coded form are given below as Equations (2)–(6).

Yield of FAd = 72.79 + 2.23X_1_ − 7.29X_2_ + 0.97X_3_ − 2.51X_1_X_3_ − 6.13X_1_^2^ − 11.24X_2_^2^ − 5.07X_3_^2^ (2)

Yield of *p*-CA = 76.72 + 5.76X_1_ − 13.18X_2_ + 3.06X_3_ + 4.17X_1_X_2_ − 10.83X_2_^2^ (3)

Yield of 3-gluK = 128.60 + 23.60X_1_ − 35.20X_2_ − 34.37X_2_^2^ (4)

Yield of EA = 278.25 + 19.08X_1_ − 150.04X_2_ + 13.88X_3_ − 20.17X_2_X_3_ − 90.94X_2_^2^ (5)

Ln Yield of tiliroside = 7.46 + 0.28X_1_ − 1.06X_2_ + 0.05X_3_ + 0.04X_2_X_3_ − 0.13X_1_^2^ − 0.98X_2_^2^ (6)

Response variables were positively correlated with temperature (X_1_) and the number of extraction cycles (X_3_) but negatively correlated with solvent ratio (X_2_) and with the quadratic term of solvent ratio (X_2_^2^). The rest of the statistically significant independent variables (their interactions and the quadratic terms) were at different correlations, depending on the chemical compound. Table 4 shows the optimal parameters for each polyphenol considered separately. Differences in the values of these parameters result from the natural and chemical structure of a given polyphenol compound. For example, the optimal extraction temperature for the ferulic acid derivative is the lowest among all analyzed polyphenols, and the highest concentration of ethanol in the (water/ethanol) configuration is in the case of ellagic acid. This may also be related to the fact that polyphenols may occur in complex forms with proteins, carbohydrates, and other insoluble phenols with high molecular mass [48], which also affects the differences in the obtained parameters.

Considering all variables, such as extraction temperature, solvent ratio, and extraction cycles, the optimal extraction conditions for the examined compound yield are given in Table 4.

### 2.2. Chromatographic Analysis and Isolation

UHPLC-DAD-MS method was employed to analyze the extracts from defatted strawberry seeds. Examples of chromatograms obtained using spectrophotometric and mass detection are presented in Figure S1 (Supplementary Materials). Data used for identification are included in Table S1 and Figure S2.

The qualitative composition of ASE extracts was in accordance with data in the literature [30]. Nine phenolic compounds were identified with two predominant components, namely ellagic acid and tiliroside. The obtained model has been experimentally verified, taking into consideration the maximal tiliroside yield as a target component for further isolation. The established ASE conditions allowed the extraction of 2431.5 ± 28.9 µg tiliroside from one gram of plant material (243.2 mg/100 g). It should be noted that ASE efficacy was significantly higher than ultrasonic or heat reflux extraction used in our previous study [22], and the tiliroside yield was approximately two-fold higher than for the ultrasonic-assisted process.

To ensure effective isolation, the ratio of the target component to the other components of the extract should be appropriately high. Therefore, to reduce the polar matrix of the defatted strawberry seeds, preliminary ASE extraction with hot water was applied as a first step before isolating the tiliroside-containing fraction. It allowed for the increase in the ratio of tiliroside to the matrix in the extract (Figure S3). Such a tiliroside-rich fraction was further subjected to preparative chromatography (Figures S4 and S5). The procedure allows for obtaining tiliroside with 95.2% purity relatively easily (the calculation was based on the tiliroside standard calibration curve).

It should be highlighted that the isolation of tiliroside usually proceeds in several stages of solvent–solvent extraction to reduce the matrix, which hampers the chromatographic process [13,49]. Isolation from defatted strawberry seeds allows for the elimination of many stages, and the optimized ASE procedure provides a product with high efficiency.

## 3. Materials and Methods

### 3.1. Plant Material

The research material consisted of strawberry seeds, which were purchased from a domestic supplier (Zielony Klub, Kielce, Poland). Before scCO_2_ extraction, the seeds were crushed using a Sipma H-752 roller crusher (Lublin, Poland). After crushing, the plant material was weighed and placed in the extractor. The weight of the charge to the extractor was 4350 g. The scCO_2_ process was carried out in continuous mode at a CO_2_ flow rate of 146 kg/h and with a total extraction time of 120 min at a temperature of 40 °C and a pressure of 230 bar in a quarter-technical installation [50] in Analytical Department Łukasiewicz Research Network, New Chemical Syntheses Institute, in Puławy (Puławy, Poland). The defatted seed residues obtained in this way were stored at 4 °C until polyphenol analysis

### 3.2. Extraction

The obtained defatted residue was extracted using ASE 350 (Accelerated Solvent Extractor, Dionex Corporation, Sunnyvale, CA, USA). The 3.00 g sample was placed in 10 mL stainless steel reaction cells containing filter at the bottom to reduce the presence of suspended particles in the receiver vials. The process was carried out in several variants of extraction parameters, which allowed the establishment of the relationship between the content of individual biologically active compounds in the extract (output/dependent variables) and the indicated process parameters (input/independent variables).

The ASE process ran at constant parameters as follows: pressure—1500 psi; oven preheat—5 min; cell heat—5 min; extraction static time—7 min; rinse volume with clean solvent—60%; and purge—60 s. Temperature, solvent ratio (water to 96% ethanol), and the number of static cycles were the independent variables. The duration of ASE extraction in individual experiments ranged from 30 to 50 min. After extraction, the volume of the obtained extracts was measured, and, until analysis, the samples were stored in amber glass bottles, protected from light at a temperature of 5 °C.

The exact design of the experiments, performed in random order to avoid systematic errors, is shown in Table 5.

### 3.3. Chromatography

#### 3.3.1. Ultra-High-Performance Liquid Chromatography (UPLC)

Solvents and standards were from Sigma-Aldrich (Saint Louis, MO, USA). Chromatography was carried out using an ultra-high-performance liquid chromatography (UPLC) Infinity Series II combined with MS with electrospray ionization (ESI) and DAD detector (Agilent Technologies Santa Clara, CA, USA) and Titan column (10 cm × 2.1 mm, 1.9 µm) (Supelco, Sigma-Aldrich, USA). The eluent was composed of water (A) and acetonitrile (B), both acidified with 0.05% formic acid. The elution gradient program and MS working parameters were detailed and described in the previous work [22].

#### 3.3.2. Preparative Chromatography (pLC)

Tiliroside-rich fraction obtained using ASE-optimized conditions was concentrated and subjected to LC separation. The isolation of tiliroside was performed using a preparative chromatograph with a UV-Vis detector, operating within a wavelength range of 200–400 nm (VWR^®^ LaPrep Sigma, Merck). Chromolith^®^ SemiPrep RP-18 end-capped 100-10 monolithic column was applied for separation using methanol and water (75:25, *v*/*v*) as eluent. The identity of isolated compound was confirmed by comparison of the mass spectrum and UV-Vis spectrum with tiliroside standard.

### 3.4. Statistical Analysis

Two independent experiments were conducted for each experimental condition. The values measured were expressed as mean ± standard deviation (SD). The experimental design and statistical analysis were performed using Microsoft Excel 2013 and Design Expert 11.0.6.0 (Stat-Ease Inc., Minneapolis, MN, USA) programs. The analysis of variance (ANOVA) was performed to assess the significance of the estimated coefficients in the model. The test of statistical significance was based on error criteria with a confidence level of 95%. To optimize the extraction process, the method of statistical experimental planning and the Box–Behnken design were selected, obtaining response surface graphs and polynomial equations/models. The influence of the selected parameters on the response variables was approximated by a second-order polynomial regression model described by Equation (7):

(7) $$y=\beta_{0}+\beta_{1}X_{1}+\beta_{2}X_{2}+\beta_{3}X_{3}+\beta_{12}X_{1}X_{2}+\beta_{13}X_{1}X_{3}+\beta_{23}X_{2}X_{3}+\beta_{11}X_{1}^{2}+\beta_{22}X_{2}^{2}+\beta_{33}X_{3}^{2}$$

where *y* denotes the response variable; *X*_1_, *X*_2_, and *X*_3_ denote independent variables, which are, respectively, temperature, solvent ratio, and extraction cycles; and *β* denotes coefficients in the equation that define the equation constant (*β*_0_), main effects of parameters (*β*_1_, *β*_2_, *β*_3_), their interaction (*β*_12_, *β*_23_, *β*_13_), and quadratic terms (*β*_11_, *β*_22_, *β*_33_).

The coefficient of determination *R*^2^, adjusted coefficient of determination *R*^2^, predicted coefficient *R*^2^, and the lack of fit were used to evaluate the accuracy of adjusting the experimental data to the model.

## 4. Conclusions

ASE combined with Box–Behnken design allowed for the extraction of polyphenolic fractions from plant material with high effectiveness. All obtained mathematical models generally showed an increase in the efficiency of obtaining the polyphenolic compounds with an increase in temperature, ethanol content, and the number of extraction cycles. However, in several of the compounds discussed, the yield began to decrease when the ethanol content exceeded approximately 60% (the ferulic acid derivative and *p*-coumaric acid) and 65% (kaempferol 3-glucoside and tiliroside). Also, the number of extraction cycles significantly influenced the yield of *p*-coumaric and ellagic acid, whereas, in the case of ferulic acid derivative, the interaction effects between the temperature and the number of cycles had a significant effect on yield. When the temperature was more than 60 °C and the number of extraction cycles was more than 3, the yield of ferulic acid derivative began to reduce. In summary, taking into account tiliroside, which was the target component, the best results were obtained under the following optimal conditions: four extraction cycles, a temperature of 65 °C, and an ethanol percentage (in a water–ethanol mixture) of 63%.

Furthermore, defatted strawberry seeds proved to be a convenient source of tiliroside because the matrix of accompanying components is relatively poor, which facilitates separation.

## Figures and Tables

**Table 1:** Experimental matrix and results in the BBD: X_1_—temperature; X_2_—solvent ratio; X_3_—extraction cycles. Data are given as the mean values in µg/g of plant material ± SD.

Run Order	Independent Variables			Response Variables					
	X_1_	X_2_	X_3_	FAd	*p*-CA	3-gluK	EA	Tiliroside	Yield (%)
1	0	0	0	74.67 ± 1.70	83.10 ± 2.22	134.80 ± 2.71	257.44 ± 1.20	1757.4 ± 32.1	5.41 ± 0.07
2	−1	0	1	62.49 ± 0.75	72.33 ± 1.13	101.88 ± 2.88	280.55 ± 1.52	1196.0 ± 45.6	5.40 ± 0.02
3	1	0	−1	65.70 ± 0.69	77.57 ± 1.50	157.52 ± 3.60	312.61 ± 2.38	1948.1 ± 8.8	5.58 ± 0.05
4	−1	0	−1	55.28 ± 1.68	69.49 ± 1.16	102.84 ± 0.29	260.21 ± 1.75	1085.3 ± 24.6	5.31 ± 0.10
5	0	1	1	50.44 ± 0.79	59.47 ± 0.82	66.67 ± 1.25	41.28 ± 2.26	252.32 ± 4.87	6.22 ± 0.13
6	0	0	0	72.84 ± 1.41	75.65 ± 4.94	128.50 ± 1.69	267.20 ± 3.46	1766.1 ± 14.2	5.48 ± 0.02
7	1	0	1	62.88 ± 2.32	78.84 ± 0.98	151.83 ± 6.94	308.69 ± 3.32	2064.7 ± 36.3	5.65 ± 0.09
8	0	1	−1	51.50 ± 1.69	44.36 ± 1.49	46.35 ± 3.42	34.29 ± 1.26	205.75 ± 17.3	5.71 ± 0.06
9	0	−1	1	64.22 ± 0.51	81.03 ± 0.44	143.84 ± 1.56	381.02 ± 11.7	1840.5 ± 5.4	5.10 ± 0.07
10	0	−1	−1	59.76 ± 0.20	75.81 ± 1.37	121.50 ± 0.14	293.37 ± 2.76	1775.9 ± 32.3	4.85 ± 0.18
11	0	0	0	70.85 ± 2.39	80.09 ± 1.13	122.82 ± 4.01	261.01 ± 1.62	1805.3 ± 14.4	5.53 ± 0.04
12	1	−1	0	65.45 ± 1.08	83.43 ± 1.24	143.37 ± 1.64	367.91 ± 3.20	2230.1 ± 19.3	5.25 ± 0.04
13	1	1	0	48.93 ± 6.07	65.57 ± 1.32	86.48 ± 3.40	42.36 ± 1.26	264.35 ± 6.91	6.10 ± 0.16
14	−1	1	0	43.79 ± 1.81	41.48 ± 1.53	36.64 ± 3.69	31.13 ± 1.16	145.8 ± 5.29	5.75 ± 0.11
15	−1	−1	0	63.53 ± 2.63	76.02 ± 2.49	109.01 ± 11.9	307.05 ± 17.8	1353.4 ± 19.4	4.67 ± 0.13

FAd—ferulic acid derivative; *p*CA—*p*-coumaric acid; 3-gluK—kaempferol 3-glucoside; EA—ellagic acid.

**Table 2:** Regression coefficients of the full quadratic models with their statistical significance and model parameters for investigated polyphenols.

Source	FAd		*p*-CA		3-gluK		EA		Tiliroside	
	β	*p*-Value	β	*p*-Value	β	*p*-Value	β	*p*-Value	β	*p*-Value
Model		0.0026		0.0055		0.0018		<0.0001		0.0001
Lack of Fit		0.3003		0.4017		0.2095		0.0876		0.0275
β_0_	72.79		79.61		128.71		261.88		1776.29	
X_1_	2.23	0.0612	5.76	0.0140	23.60	0.0011	19.08	0.0084	340.85	0.0005
X_2_	−7.29	0.0005	−13.18	0.0004	−35.20	0.0002	−150.04	<0.0001	−791.46	<0.0001
X_3_	0.97	0.3425	3.06	0.1067	4.50	0.2566	13.88	0.0281	42.31	0.3606
X_1_X_2_	0.80	0.5676	4.17	0.1164	3.87	0.4716	−12.41	0.1110	−189.56	0.0244
X_1_X_3_	−2.51	0.1147	−0.39	0.8648	−1.18	0.8215	−6.06	0.3880	1.47	0.9813
X_2_X_3_	−1.38	0.3405	2.47	0.3123	−0.50	0.9232	−20.17	0.0256	−4.49	0.9428
X_1_^2^	−6.13	0.0065	−1.80	0.4680	−0.45	0.9335	14.13	0.0880	−111.48	0.1317
X_2_^2^	−11.24	0.0004	−11.19	0.0045	−34.38	0.0012	−88.90	<0.0001	−666.39	0.0001
X_3_^2^	−5.07	0.0138	−3.25	0.2145	0.26	0.9614	14.50	0.0819	−91.28	0.2004
R^2^		0.9704		0.9598		0.9747	0.9963			0.9910
Adjusted R^2^		0.9172		0.8874		0.9290	0.9895			0.9747
Predicted R^2^		0.6131		0.5167		0.6450	0.9432			0.8576

FAd—ferulic acid derivative; *p*CA—*p*-coumaric acid; 3-gluK—kaempferol 3-glucoside; EA—ellagic acid; *p* < 0.0001 very highly significant, *p* < 0.01 very significant, *p* < 0.05 significant.

**Table 3:** Regression coefficients of the reduced models with their statistical significance and model parameters for investigated polyphenols.

Source	FAd		*p*-CA		3-gluK		EA		Tiliroside *	
	β	*p*-Value	β	*p*-Value	β	*p*-Value	β	*p*-Value	β	*p*-Value
Model		0.0002		0.0001		<0.0001		<0.0001		<0.0001
Lack of Fit		0.3598		0.4685		0.8357		0.0560		0.0842
β_0_	72.79		76.72		128.60		278.25		7.46	
X_1_	2.23	0.0411	5.76	0.0046	23.60	<0.0001	19.08	0.0160	0.28	<0.0001
X_2_	−7.29	0.0001	−13.18	<0.0001	−35.20	<0.0001	−150.04	<0.0001	−1.06	<0.0001
X_3_	0.97	0.3121	3.06	0.0779			13.88	0.0597	0.05	0.0106
X_1_X_2_			4.17	0.0870						
X_1_X_3_	−2.51	0.0878								
X_2_X_3_							−20.17	0.0543	0.04	0.0807
X_1_^2^	−6.13	0.0023							−0.13	0.0004
X_2_^2^	−11.24	0.0001	−10.83	0.0010	−34.37	<0.0001	−90.94	<0.0001	−0.98	<0.0001
X_3_^2^	−5.07	0.0052								
R^2^		0.9617		0.9294		0.9629	0.9864			0.9989
Adjusted R^2^		0.9233		0.8902		0.9528	0.9788			0.9981
Predicted R^2^		0.8479		0.8044		0.9305	0.9611			0.9943

FAd—ferulic acid derivative; *p*CA—*p*-coumaric acid; 3-gluK—kaempferol 3-glucoside; EA—ellagic acid; *p* < 0.0001 very highly significant, *p* < 0.01 very significant, *p* < 0.05 significant; * results after additional transformation (ln (Y)).

**Table 4:** The optimal ASE conditions for investigated polyphenols.

Compound	Temperature, °C	Ratio H_2_O:EtOH	Extraction Cycles
ferulic acid deriv.	57	42:58	3
*p*-coumaric acid	65	40:60	4
kaempferol 3-glucoside	65	37:63	4
ellagic acid	65	27:73	4
tiliroside	65	37:63	4

**Table 5:** Experimental matrix of the Box–Behnken design with coded and uncoded independent variables.

Run Order	Coded Independent Variables			Uncoded Independent Variables		
	X_1_	X_2_	X_3_	Temperature (°C)	Solvent Ratio (H_2_O:EtOH)	Extraction Cycles
1	0	0	0	55	50:50	3
2	−1	0	1	45	50:50	4
3	1	0	−1	65	50:50	2
4	−1	0	−1	45	50:50	2
5	0	1	1	55	75:25	4
6	0	0	0	55	50:50	3
7	1	0	1	65	50:50	4
8	0	1	−1	55	75:25	2
9	0	−1	1	55	25:75	4
10	0	−1	−1	55	25:75	2
11	0	0	0	55	50:50	3
12	1	−1	0	65	25:75	3
13	1	1	0	65	75:25	3
14	−1	1	0	45	75:25	3
15	−1	−1	0	45	25:75	3

## Data Availability

The data presented in this study are available on request from the corresponding author.

## Supplementary Materials

The following supporting information can be downloaded at https://www.mdpi.com/article/10.3390/molecules29133051/s1. Table S1. Data used for identification of main polyphenolic constituents extracted from defatted strawberry seeds; Figure S1: Example of base peak chromatogram (BPC) in negative ionization mode (red line) and DAD chromatogram shown at a wavelength of 254 nm (green line) and 320 nm (blue line) for the extract from defatted strawberry seeds obtained using accelerated solvent extraction; Figure S2. MS spectra of compounds identified in the extracts from defatted strawberry seeds obtained using accelerated solvent extraction. Names of the compounds are given in Table S1; Figure S3. Overlapped chromatograms of the extracts obtained using pretreatment of plant material with water (red line) and without pretreatment (blue line); Figure S4. Chromatogram obtained using preparative monolithic column with marked region of isolation. 1 –tiliroside; Figure S5. Chromatogram registered at 320 nm (blue line) and at 245 nm (red line) (a) and base peak chromatogram (c) of tiliroside isolated from plant material; Figure S6. UV-Vis and MS spectrum of the tiliroside standard (grey line) and the isolated compound (blue line).
