# Supplementary material for: Optimizing the Extraction of the Polyphenolic Fraction from Defatted Strawberry Seeds for Tiliroside Isolation Using Accelerated Solvent Extraction Combined with a Box–Behnken Design
Source: Molecules. 2024 Jun 27;29(13):3051. doi: 10.3390/molecules29133051 (PMC11243754; doi:10.3390/molecules29133051)
Supplement: Supplementary file 1 [file molecules-29-03051-s001.zip › molecules-3039728-supplementary.pdf]

Optimizing the Extraction of the Polyphenolic Fraction from Defatted Strawberry Seeds for Tiliroside Isolation Using Accelerated Solvent Extraction Combined with a Box–Behnken Design

Magdalena Wójciak [1,*], Barbara Mazurek [2], Weronika Wójciak [1], Dorota Kostrzewa [2], Magdalena Żuk [1], Mariusz Chmiel [2], Tomasz Kubrak [3] and Ireneusz Sowa [1]

1 Department of Analytical Chemistry, Medical University of Lublin, Chodźki 4a, 20-093 Lublin, Poland; weronikawojciak01@gmail.com (W.W.); magdalena.zu25@gmail.com (M.Ż.); ireneusz.sowa@umlub.pl (I.S.)
2 Analytical Department, Łukasiewicz Research Network—New Chemical Syntheses Institute, Aleja Tysiąclecia Państwa Polskiego 13a, 24-110 Puławy, Poland; barbara.mazurek@ins.lukasiewicz.gov.pl (B.M.); dorota.kostrzewa@ins.lukasiewicz.gov.pl (D.K.); mariusz.chmiel@ins.lukasiewicz.gov.pl (M.C.)
3 Department of Biochemistry and General Chemistry, Medical College of The University of Rzeszów, Rzeszów, 2A Kopisto St., 35-959 Rzeszów, Poland; tkubrak@ur.edu.pl
* Correspondence: magdalena.wojciak@umlub.pl

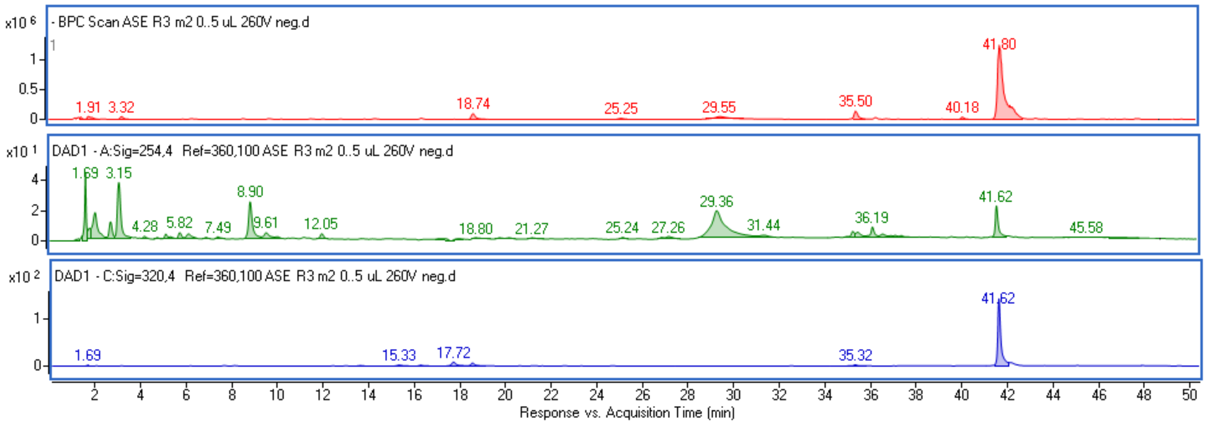

Figure S1. Example of base peak chromatogram (BPC) in negative ionization mode (red line) and DAD chromatogram shown at a wavelength of 254 nm (green line) and 320 nm (blue line) for the extract from defatted strawberry seeds obtained using accelerated solvent extraction.

Table S1. Data used for identification of main polyphenolic constituents extracted from defatted strawberry seeds.

	R_T (min)	**[M-H]-**	**ppm**	**Formula**	**Name**
1	9.01	451.12512 (289)	1.18	$C_{21}H_{24}O_{11}$	catechin hexoside
2	11.61	577.13559 (289)	0.76	$C_{30}H_{26}O_{12}$	procyanidin
3	18.01	-(119,161)	-	$C_9H_8O_3$	p-coumaric acid[1]
4	18.86	679.20999 (517.193)	1.30	$C_{28}H_{39}O_{19}$	3-O-feruloylsucrose derivative
5	29.55	300.99908	0.30	$C_{14}H_6O_8$	ellagic acid[1]
6	32.11	447.05871 (301)	4.04	$C_{20}H_{16}O_{12}$	ellagic acid rhamnoside
7	34.16	447.09458 (284)	2.89	$C_{21}H_{20}O_{11}$	kaempferol glucoside[1]
8	36.46	461.07369 (315,301)	2.47	$C_{21}H_{18}O_{12}$	methylellagic acid hexoside
9	41.80	593.13221 (284)	3.61	$C_{30}H_{26}O_{13}$	tiliroside[1]

1 components were confirmed by comparison with standard

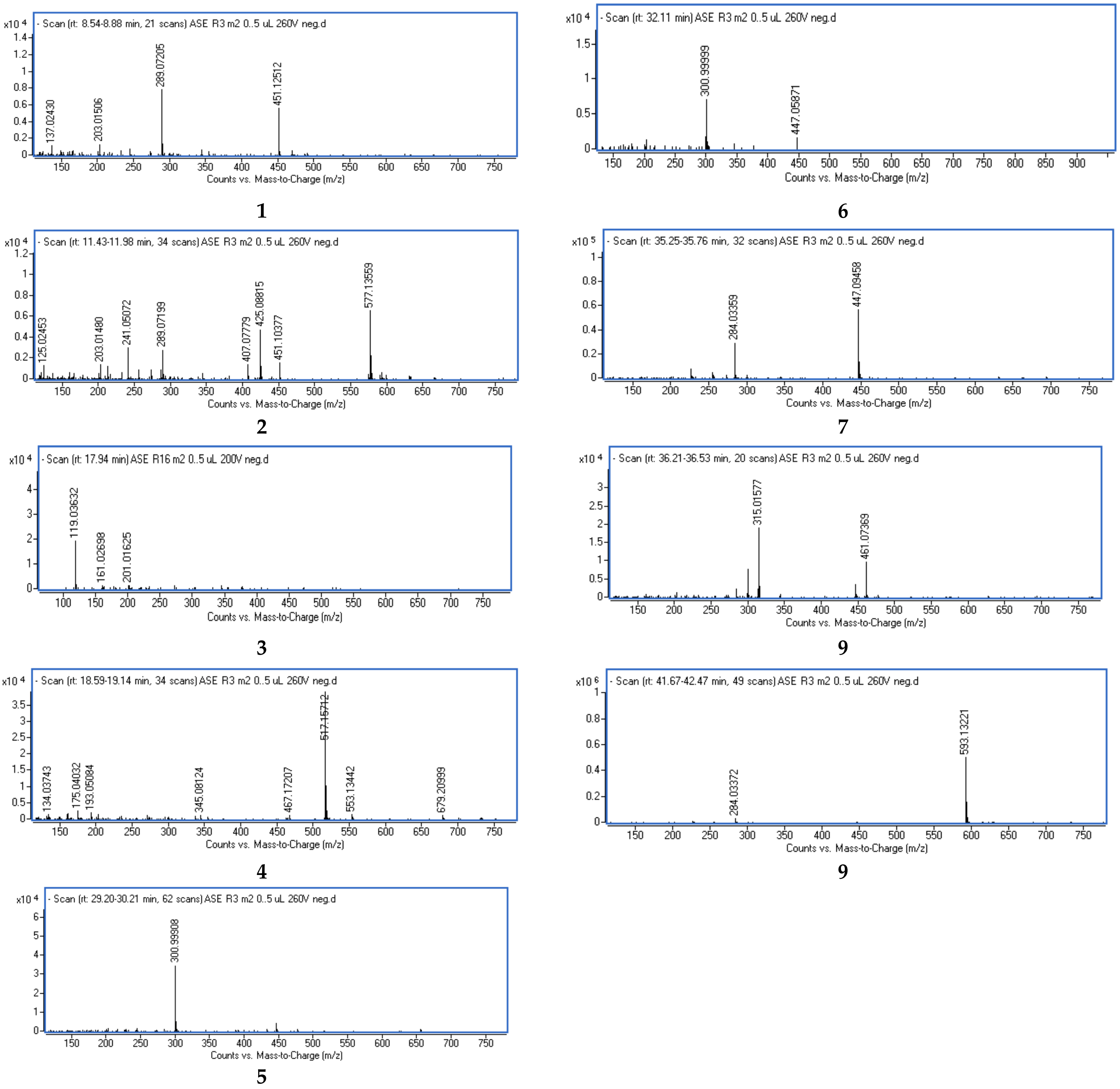

Figure S2. MS spectra of compounds identified in the extracts from defatted strawberry seeds obtained using accelerated solvent extraction. Names of the compounds are given in Table S1.

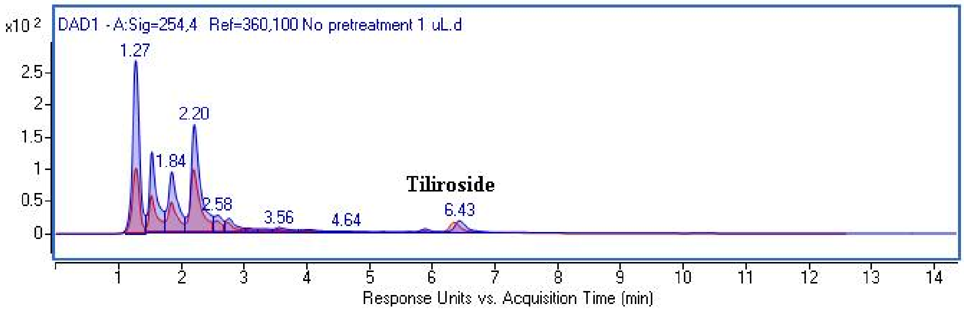

Figure S3. Overlapped chromatograms of the extracts obtained using pretreatment of plant material with water (red line) and without pretreatment (blue line).

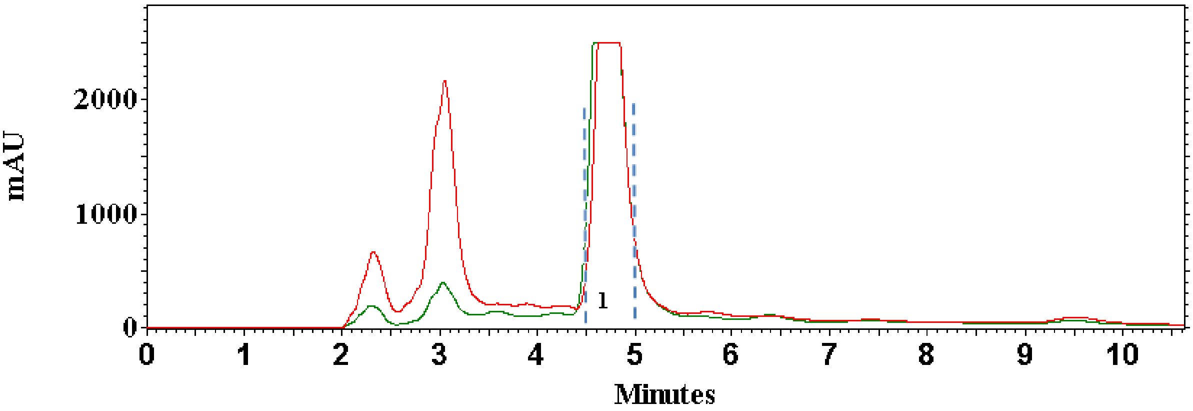

Figure S4. Chromatogram obtained using preparative monolithic column with marked region of isolation. 1 – tiliroside.

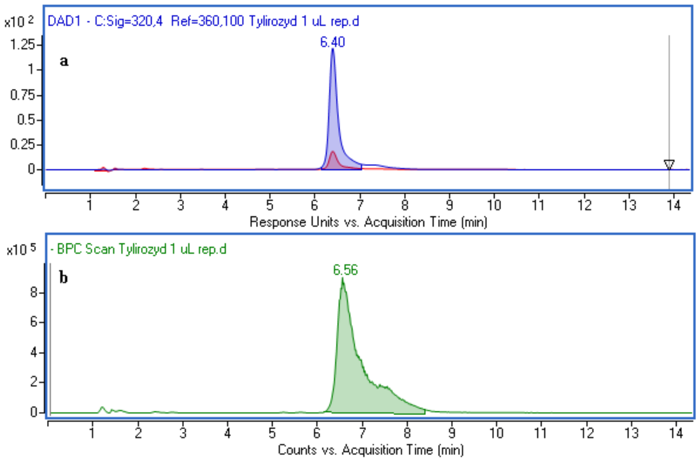

Figure S5. Chromatogram registered at 320 nm (blue line) and at 245 nm (red line) (a) and base peak chromatogram (c) of tiliroside isolated from plant material.

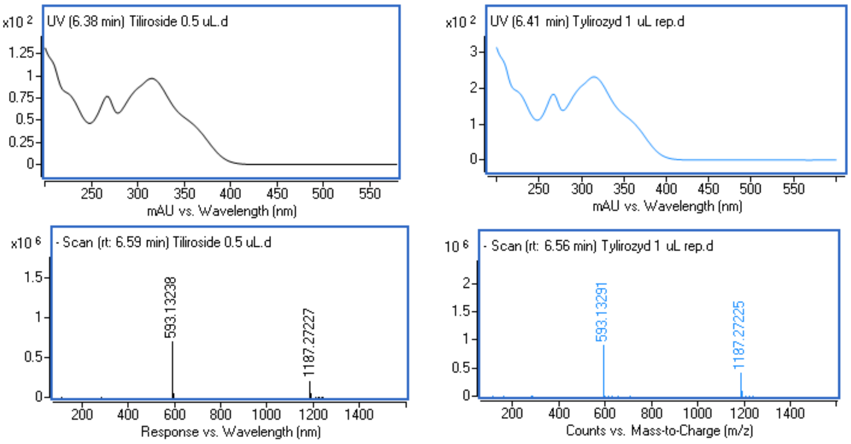

Figure S6. UV-Vis and MS spectrum of the tiliroside standard (grey line) and the isolated compound (blue line).
